# Rocuronium Can Trigger a Hypertensive Crisis in a Patient With Paraganglioma: A Case Report

**DOI:** 10.7759/cureus.59868

**Published:** 2024-05-08

**Authors:** Tomoaki Itaya, Shunichi Takagi, Takuya Saito, Takahiro Suzuki

**Affiliations:** 1 Department of Anesthesiology, Nihon University School of Medicine, Tokyo, JPN

**Keywords:** general anesthesia, propofol, remimazolam, noradrenaline, pheochromocytoma, paraganglioma, rocuronium, hypertensive crisis

## Abstract

We present a case of rocuronium-induced hypertensive crises that occurred twice in a patient with paraganglioma. An 86-year-old woman was first scheduled for laminectomy for lumbar spinal stenosis. Five minutes after intravenous induction of anesthesia using fentanyl, propofol, rocuronium, and remifentanil, the patient’s blood pressure (BP) and heart rate (HR) suddenly increased with no stimuli. Surgery was postponed because the patient was suspected of having pheochromocytoma. After that, paraganglioma was diagnosed, and surgery for removal of the paraganglioma was scheduled after the commencement of alpha-blocker therapy. The patient’s hemodynamic parameters remained stable when anesthesia was induced with an infusion of remimazolam. Subsequently, immediately after rocuronium was administered as an intravenous bolus, the patient’s arterial BP and HR increased, and plasma concentrations of noradrenaline and rocuronium had markedly increased. Ten minutes after the administration of rocuronium, the patient’s BP and HR gradually and fully recovered without any intervention. The plasma concentrations of both noradrenaline and rocuronium also concurrently decreased. We conclude that simultaneous increases in BP, HR, and plasma concentration of noradrenaline revealed a direct correlation with rocuronium.

## Introduction

The annual incidence of pheochromocytomas and paragangliomas in the general population is 3-8 per million persons per year one. Depending on the type of pheochromocytoma, it may secrete norepinephrine and epinephrine, whereas if certain paragangliomas secrete hormones, it is usually norepinephrine. The resultant alpha-adrenergic receptor stimulation causes vasoconstriction, hypovolemia, and hypertension. Hypertensive crises caused by massive catecholamine surges are a potentially life-threatening complication in these patients. Such hypertensive crises can be triggered by psychological stress (e.g., anxiety, pain, or excitation), mechanical stress (e.g., palpation of tumors, defecation, coitus, or parturition), and drugs (e.g., glucocorticoids, sympathomimetics, anticholinergic drugs, and catecholamine-sensitizing anesthetics) [1].

Undiagnosed pheochromocytomas reportedly have a high intraoperative mortality rate, and it is recommended that surgery be canceled if a hypertensive crisis is suspected during the induction of anesthesia [2]. Several cases of undiagnosed pheochromocytomas presenting with hypertensive crises after anesthesia induction have been reported [3-5]. In the previous reports, however, multiple anesthetic agents during anesthesia induction prevented the identification of rocuronium as the cause of the hypertensive crisis, and the correlation between plasma concentrations of rocuronium and noradrenaline was unknown. We report a case of hypertensive crisis after anesthesia induction in a case of paraganglioma that was likely precipitated by rocuronium. This is the first report clearly showing a correlation between an increase in blood concentrations of rocuronium and noradrenaline.

## Case presentation

Written informed consent for this publication was obtained from the patient. An 86-year-old, 56 kg woman was scheduled for laminectomy for lumbar spinal stenosis. The patient’s medical history was significant for hypertension, which was under treatment with 40 mg nifedipine and 40 mg telmisartan daily. The preoperative assessment showed no restriction of cardiac and pulmonary capacity. The patient’s blood pressure (BP) and heart rate (HR) usually ranged from 140-160/70-80 mmHg and 50-70 beats per minute (bpm), respectively. When the patient entered the operating room, her BP and HR were 181/75 mmHg and 62 bpm, respectively. The patient was preoxygenated with 100% oxygen via a face mask, followed by intravenous induction of anesthesia using fentanyl 100 µg, propofol 50 mg, rocuronium 30 mg, and remifentanil 1.5 mg/hour. Although the patient’s lungs were easily ventilated via a mask with no other stimuli to the patient, five minutes after induction of anesthesia, the patient’s BP and HR suddenly increased to 268/144 mmHg and 124 bpm, respectively. Suspecting a hypertensive crisis, intravenous administration of propofol 30 mg and inhalation of 8% sevoflurane were first added to lower BP. We suspected a hypertensive crisis caused by catecholamines released for some reasons and judged to wait for a gradual decrease without vasodilators. The patient continued to ventilate through the mask, and her BP and HR gradually decreased within the next 10 minutes and returned almost to baseline levels. However, surgery was postponed due to the possibility that the hypertensive crisis might have been due to an undiagnosed pheochromocytoma.

**Figure 1:**
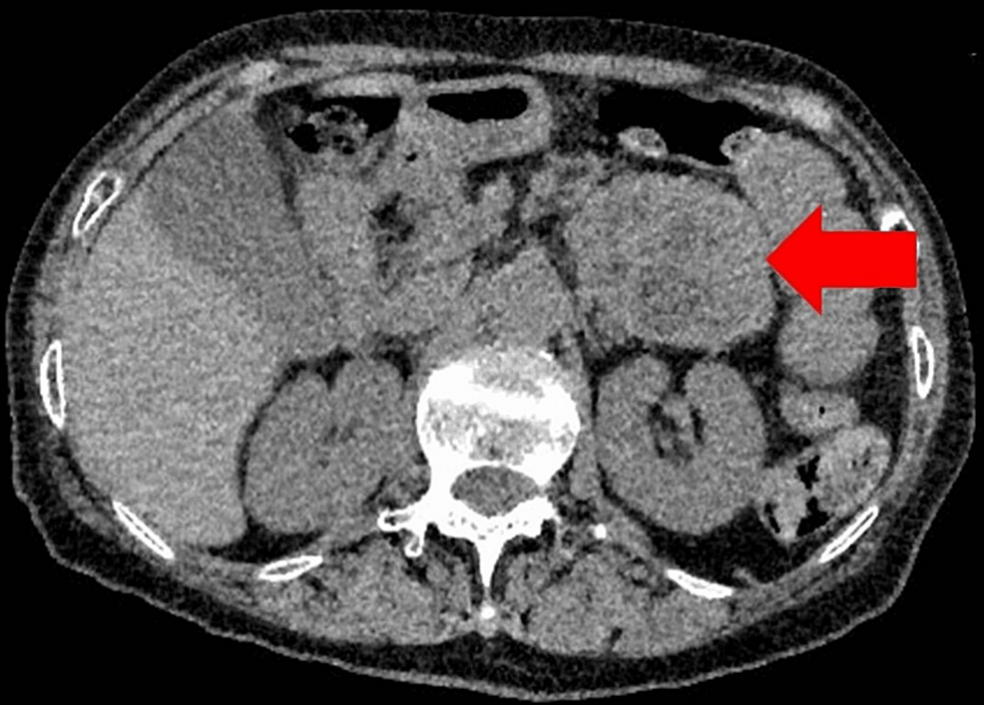
Computed tomography scan 60 x 40 mm solid mass with calcification arising from the pancreatic tail.

**Figure 2:**
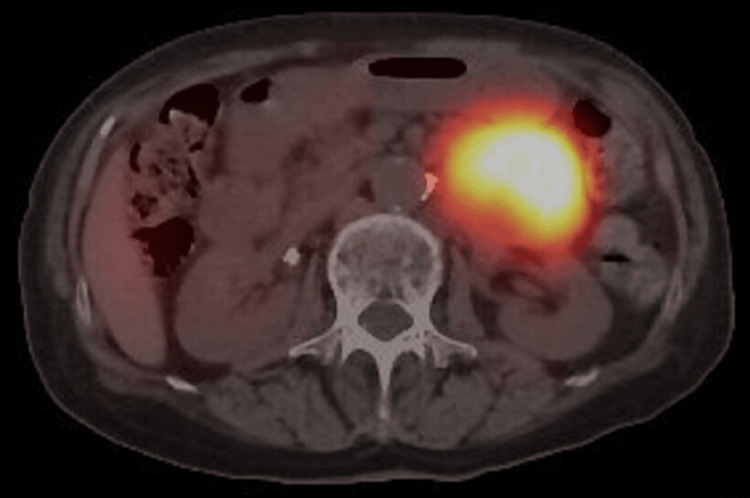
Meta-iodobenzylguanidine scintigraphy scan Pancreatic tail paraganglioma without metastasis.

Subsequently, a computed tomography (CT) scan revealed a 60 x 40 mm solid mass with calcification arising from the pancreatic tail (Figure 1). The tumor was suspected as a paraganglioma because 24-hour urine (collected while the patient was admitted to the hospital), vanillylmandelic acid, normetanephrine, and noradrenaline levels were found to be elevated to 24.5 mg (reference: 0-6 mg), 2.46 mg (reference: 0-0.3 mg), and 356 µg (reference: 31-160 µg), respectively. A meta-iodobenzylguanidine scintigraphy scan confirmed a pancreatic tail paraganglioma without metastasis (Figure 2). Surgery for removal of the paraganglioma was scheduled after the commencement of alpha-blocker therapy (16 mg doxazosin daily). During the interim period, the patient’s weight decreased to 41 kg since excess catecholamine might stimulate heat production in brown adipose tissue and improve the patient’s metabolism.

**Figure 3:**
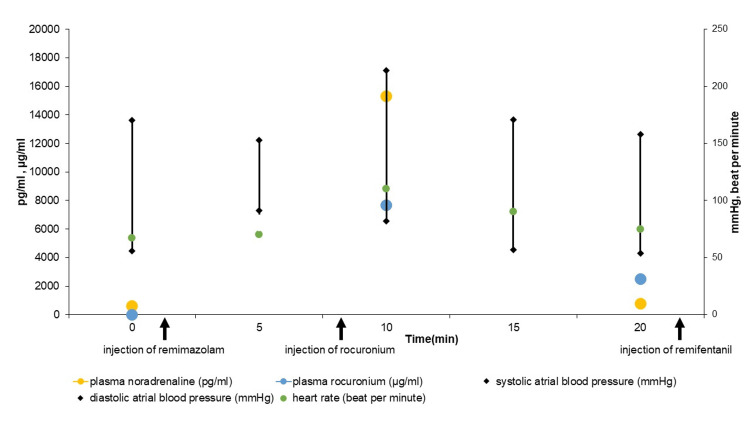
Key events timeline Blood pressure, heart rate, blood concentration of rocuronium, blood concentration of noradrenaline after control, injection of remimazolam, injection of rocuronium, and injection of remifentanil.

The patient was informed about the possibility of a hypertensive crisis caused by rocuronium, and consent to accept rocuronium was obtained because there are no other available nondepolarizing neuromuscular blocking agents in Japan. After being admitted to the operation room, a 20-gauge catheter was inserted into the patient’s left radial artery at the wrist under local anesthesia for continuous invasive arterial BP (ABP) monitoring and blood samples to measure the plasma concentration of noradrenaline and rocuronium by high-performance liquid chromatography. At that time, the patient’s ABP and HR were 170/56 mmHg and 67 bpm, respectively, and the plasma concentration of noradrenaline was 610 pg/mL (reference: 100-450 pg/mL). Under preoxygenation with 100% oxygen via a face mask, anesthesia was induced with an infusion of remimazolam (82 mg/hour). The patient lost consciousness three minutes after anesthesia induction with an effect site concentration of 0.19 µg/mL [6], during which time the patient’s hemodynamic parameters remained stable, with an ABP of 161/53 mmHg and HR of 73 bpm, along with adequate hypnotic effect, as shown by a bispectral index of 40. After confirming the ease of mask ventilation, the remimazolam infusion was reduced to 18.3 mg/hour, resulting in an effect site concentration of 0.4 µg/mL, more than twice the concentration for loss of consciousness [6]. Subsequently, immediately after 30 mg of rocuronium was administered as an intravenous bolus, the patient’s ABP and HR increased rapidly to 214/82 mmHg and 110 bpm, respectively, at which time there was no contact with the patient, and the bispectal index was 40-50. At that time, the patient’s plasma concentration of noradrenaline had markedly increased to 15,313 pg/mL, and the patient’s plasma concentration of rocuronium was 7,670 ng/mL. Ten minutes after the administration of rocuronium, the patient’s ABP and HR gradually and fully recovered to 158/54 mmHg and 75 bpm, respectively, without any intervention. The plasma concentrations of both noradrenaline and rocuronium concurrently decreased to 784 pg/mL and 2,496.6 ng/mL, respectively (Figure 3). After the patient’s ABP and HR were stabilized, a continuous infusion of remifentanil 1.3 mg/hour was started. Then, the patient’s trachea was safely intubated with an endotracheal tube. Even though tracheal intubation is an invasive procedure, the patient’s ABP and HR remained stable during intubation, at 122/42 mmHg and 64 bpm, respectively. Intraoperatively, three additional 10 mg doses of rocuronium were administered before removal of the paraganglioma, although with no further hypertensive episodes. The surgery was performed uneventfully.

**Figure 4:**
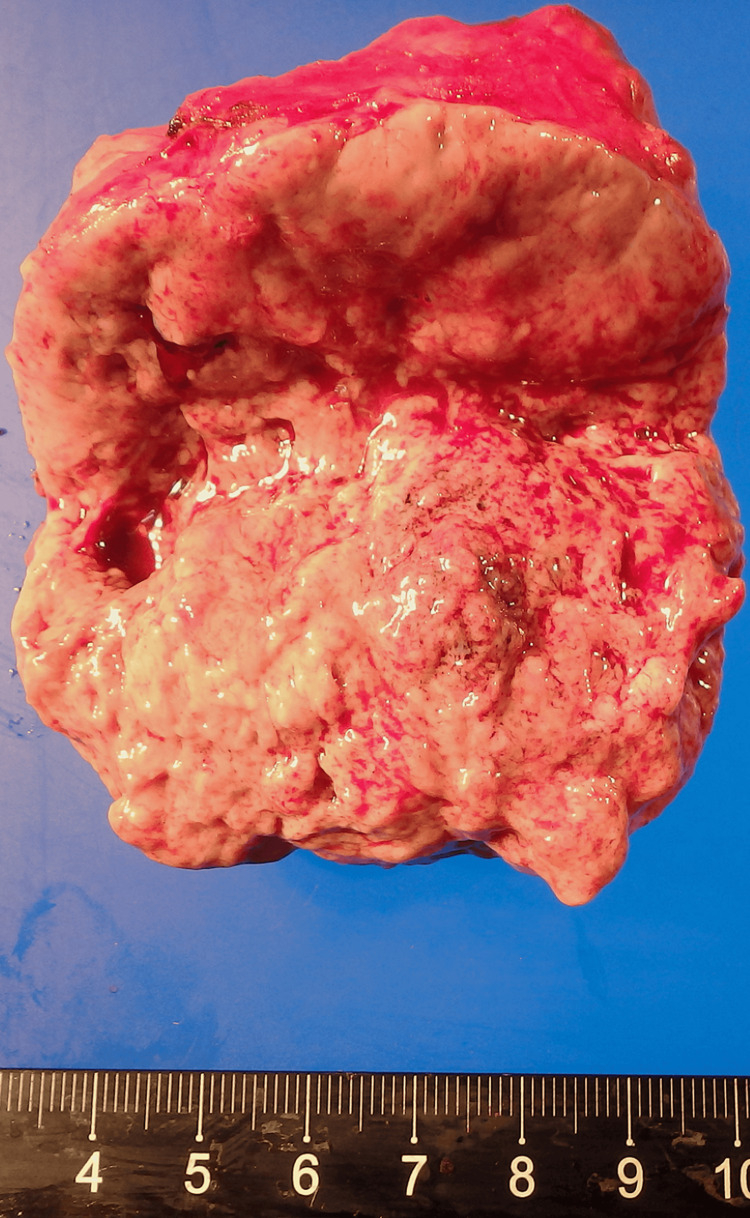
Postoperative histological evaluation 70 x 60 x 30 mm-sized paraganglioma.

Postoperative histological evaluation showed a 70 x 60 x 30 mm sized paraganglioma (Figure 4). The patient's plasma concentration of noradrenaline normalized postoperatively. The patient subsequently underwent laminectomy using rocuronium without any further hypertensive crisis.

## Discussion

In this case, the patient’s BP increased severely immediately after a bolus injection of rocuronium during both instances of general anesthesia induction. Since there was no obvious correlation between any other factor and the hypertensive crisis, we determined that rocuronium caused the hypertensive crisis in this patient with paraganglioma. Evaluation of the literature showed three previous studies that similarly reported that rocuronium can cause hypertensive crisis [3-5], although no report definitively identified rocuronium as the cause of hypertensive crisis, because of simultaneous administration of multiple anesthetic agents during anesthesia induction. Even during our first anesthesia event, the causative agent could not be identified for the same reason. During the second anesthesia, rocuronium was given separately from the other drugs to identify the presence of a correlation between rocuronium administration and the hypertensive crisis. However, despite preoperative control of hemodynamics using alpha-blockers, the patient’s plasma concentration of noradrenaline increased about 25-fold relative to its pre-rocuronium level. The patient’s BP and plasma concentration of noradrenaline decreased relatively quickly over time thereafter, mirroring the decrease in plasma rocuronium concentrations (Figure 3).

Muscarinic receptors are located in both, the atrial pacemaker and papillary muscle, and rocuronium is known to exert opposing effects for the M2 muscarinic receptors [7]. Sato et al. reported that rocuronium might increase norepinephrine release from human atrial tissue by inhibiting muscarinic receptors [8]. Although it is unclear whether this phenomenon has clinical relevance, parasympathetic depression occurs commonly in daily life. While this does not usually cause symptoms, in patients with paraganglioma, even a small amount of parasympathetic depression can upset the balance between sympathetic and parasympathetic nerves. Such an imbalance might have caused the hypertensive crisis in our patient. However, since additional small doses of rocuronium did not cause a hypertensive crisis in this patient, it is possible that only higher concentrations of rocuronium promote norepinephrine secretion from paragangliomas. Since no previous reports have examined the direct effect of rocuronium on paraganglioma cells (e.g., PC12), further research is needed to clarify this.

Rocuronium is known to induce severe burning injection pain, resulting in withdrawal movement, with a reported incidence rate of between 50% and 80% [9]. Injection pain might also precipitate a hypertensive crisis. Although our patient showed no withdrawal movement, we cannot fully deny the possibility that injection pain was a factor contributing to her hypertensive response.

Factors other than rocuronium are unlikely to have caused the hypertensive crisis in our patient for the following reasons. Psychological (e.g., excessive anxiety or excitation) and mechanical stresses (e.g., external compression of the abdomen or invasive mask ventilation) could be excluded in this patient. Additionally, the other drugs administered, including propofol, fentanyl, remifentanil, and remimazolam, are unlikely to cause a hypertensive crisis. Propofol has a sympatholytic action that significantly reduces systemic vascular resistance, cardiac contractility, and baroreceptor function. Additionally, during the second anesthesia, we choose remimazolam as the hypnotic agent to exclude propofol-induced injection pain as a cause of the hypertensive crisis. Benzodiazepines are known to inhibit dopamine release [10] and have an antihypertensive effect. Although it has been shown that fentanyl and remifentanil cause dose-dependent positive inotropic effects in the isolated rat heart [11], it is unknown whether this is of clinical significance at anesthesia induction. Additionally, both these opioids basically depress sympathetic activity. During the second anesthesia, neuromuscular blockade induced by rocuronium was once again required for the removal of the paraganglioma because rocuronium was the only available non-depolarizing muscle relaxant in Japan. However, anesthesia without rocuronium should have also been considered.

## Conclusions

We presented a case of rocuronium-induced hypertensive crisis in a patient with paraganglioma. Simultaneous measurement of plasma concentrations of rocuronium and noradrenaline revealed a direct correlation between the administration of rocuronium and expression of noradrenaline. We consider that rocuronium might cause hypertension crisis in patients with paraganglioma.
